# The Next Endoscopic Frontier: Considering a Career in Resection Endoscopy

**DOI:** 10.14309/crj.0000000000000515

**Published:** 2021-01-07

**Authors:** Neal A. Mehta

**Affiliations:** 1 Department of Gastroenterology and Hepatology, Digestive Disease and Surgery Institute, Cleveland Clinic Foundation, Cleveland, OH

## WHY ENDOSCOPIC RESECTION?

The endoscopic management of premalignant lesions and early gastrointestinal (GI) cancer is a rapidly expanding field. Pioneered in Asia and now spreading swiftly to the West, the refinement of endoscopic skill and development of endoscopic technology has allowed this subspecialty of interventional endoscopy to forge ahead in recent years with the emergence of endoscopic submucosal dissection (ESD) and endoscopic full-thickness resection (EFTR), including submucosal tunneling endoscopic resection. As the endoscopic management of early neoplasia offers a noninvasive option to spare the cost, morbidity, and mortality of surgery, oftentimes in patients who are not fit for surgical intervention, the future of resection endoscopy is bright.^1,2^

As we expand our ability to perform these complex endoscopic procedures, it is important that all GI trainees understand the indications for the endoscopic resection of premalignant and early GI neoplasms to appropriately triage patients toward the correct modality of resection. For instance, recent data have shown a rise in surgical intervention for benign colon polyps, further emphasizing the importance of incorporating endoscopic resection into current practices, avoiding surgical morbidity/mortality, and improving quality of life.^3^ For those interested in a career in resection endoscopy, comprehensive training is crucial to develop the technical skills needed to perform these higher-risk procedures. Here, we review the expanding field of resection endoscopy and the elements needed to train in this realm.

## UNDERSTANDING PATIENT SELECTION, INDICATIONS, RISKS, AND BENEFITS OF ENDOSCOPIC RESECTION

Several methods of endoscopic resection have been developed since the first therapeutic polypectomy was performed in the 1950s.^4^ Endoscopic mucosal resection (EMR) was pioneered in Japan and has led to the commonly used EMR techniques with injection or cap-suction to lift the lesion followed by snare resection.^5^ In 1988, the first attempt at ESD was performed for the en bloc resection of an early gastric tumor but it was not until 1999 that Gotoda et al published the first English description of a rectal ESD.^6,7^ Since then, ESD has flourished in Asia with established training programs. Furthermore, endoscopic technique has advanced to allow the resection of tumors arising from the muscularis propria with EFTR and submucosal tunneling endoscopic resection.^8^

Adoption of ESD and EFTR in the West has been limited because of population differences in GI cancers, longer procedure times with associated costs, and lack of established training programs. In recent years, the discrepancy in performing these procedures has started to close with small groups of Western endoscopists learning and advocating for ESD with the aid of Asian experts.^9,10^ We are now entering an era where Western trainees are starting to develop interest and learn resection endoscopic techniques without having to travel abroad.

Before the procedure, trainees are expected to understand the process of patient selection. Ideal candidates for endoscopic treatment are lesions with a negligible risk of lymph node metastasis. As the depth of tumor invasion is directly correlated with the risk of lymph node metastasis, determining this factor is critical preprocedurally.^8,11^ Hence, a firm understanding of the anatomy of the GI tract, including the layers comprising the esophagus, stomach, and colon, is critical. In addition, trainees should understand the various other factors contributing the risk of lymph node metastasis, including tumor size, lymphovascular involvement, and histology.^12^ Determination of these risk factors can be assessed with the use of cross-sectional imaging and endoscopic ultrasound (EUS). Although training in EUS is highly encouraged for practicing resection endoscopists, interpretation of EUS tissue involvement to stage GI cancers is crucial.

Given the complexity of the triage process, a multidisciplinary approach is highly recommended. The resection endoscopist works closely with the surgeon, radiologist, and oncologist to determine the best modality for resection (surgical vs endoscopic), keeping the patient's fitness and comorbidities in mind. Trainee involvement in a multidisciplinary tumor board can provide essential knowledge in triaging patients appropriately. Furthermore, it may be necessary to include other subspecialities in the planning process before resection, including anesthesia and general medicine, in case hospital admission before or after resection is needed.

Individuals interested in resection endoscopy are also expected to understand the different modalities of resection, including the risks and benefits of each option. Trainees should understand the indications for EMR, ESD, and EFTR for the esophagus, stomach, and colon as published by national and international guidelines.^13–16^ Deciding which modality of resection to choose can avoid risk and maximize benefits for the patient. For example, it is important to recognize that smaller lesions can undergo en bloc resection by EMR with minimal risk as compared to performing an ESD. However, lesions with submucosal invasion or larger lesions may benefit from ESD. Just as important as ascertaining the appropriate resection technique is planning for potential complications, including bleeding, perforation, and stricture formation. The management of periprocedural anticoagulants is particularly important. Familiarity with hemostatic clips, over-the-scope clips, coagulation graspers, and dilation devices is critical. Pre-emptive planning and training for how to manage these complications is vital and potentially life-saving.

## DEVELOPING THE TECHNICAL ENDOSCOPIC SKILLS TO PERFORM ENDOSCOPIC RESECTION

It would be prudent for a trainee interested in resection endoscopy to get involved early in their fellowship to develop the technical skills for resection. The first goal is to understand the steps of each resection procedure. Conventional ESD includes mucosal markings, submucosal injection, circumferential incision, and finally, submucosal dissection.^8,11^ EFTR includes nonexposed and exposed approaches, which can be further subdivided into tunneled and nontunneled techniques.^17^ A trainee must next learn the endoscopic tools needed to perform these procedures. This includes a variety of submucosal injection agents and electrosurgical knives. Injection needles, coagulation graspers, hemostatic clips, endoscopic caps, and various retraction devices may also be used. An understanding of the electrosurgical generator and the appropriate settings used to perform these procedures is also important. As the boundaries of resection endoscopy expand, technologic innovation has paralleled this growth. Unique devices, innovative techniques, and laparoscopic/robotic platforms are all currently being developed to make resection endoscopy easier, safer, and faster to perform.^18,19^

Finally, it is important to recognize that attaining competence, let alone mastery, of these procedures requires persistence due to the steep learning curve.^9,10^ Japanese training programs dedicate an entire year to learning endoscopic resection procedures. Given the limited training programs for resection endoscopy in the West, trainees should attempt to get early hands-on experience to accrue technical skill. This includes starting with the observation of procedures early in fellowship. Observation allows for the development of pattern recognition for various situations during the procedure. Another useful opportunity for hands-on experience during fellowship training includes animal laboratories using ex-vivo models. Repetition of the procedure on explanted animal models allows a trainee to develop endoscopic tip control and skills. If a trainee has developed sufficient skill on ex vivo models, and the opportunity is available, in vivo animal training is an option.

Ultimately, after observation and animal model training, an advanced endoscopy fellowship program should be the next step to learn the technical skill necessary to perform resection endoscopy. As most advanced endoscopy fellowship programs tend to focus on endoscopic retrograde cholangiopancreatography or EUS, the trainee will likely need to be at a program with a dedicated resection endoscopy track or with sufficient volume to obtain the basic skills to perform resection endoscopy. These skills can be further built on as an early staff. In addition, options to travel abroad to Asia for training in resection endoscopy are available as well as endoscopic workshops sponsored by national societies.

In summary, a career in resection endoscopy requires commitment but can be extremely rewarding. By providing a non-invasive curative option for patients with early cancers and premalignant lesions, these endoscopic procedures can spare many patients the morbidity and mortality of open surgery and possible allow for better quality of life. As the popularity of these procedures grows in the West and technologic advances make these procedures safer to perform, fellows should consider endoscopic resection as a career path.

## DISCLOSURES

Author contributions: N. Mehta is the sole author and is the article guarantor.

Acknowledgments: The author acknowledges Amit Bhatt, MD.

Financial disclosure: None to report.

Informed consent was obtained for this case report.
